# Synthesis, Structural Characterization, and Optical Properties of Benzene-Fused Tetracyclic and Pentacyclic Stiboles

**DOI:** 10.3390/molecules26010222

**Published:** 2021-01-04

**Authors:** Mio Matsumura, Yuki Matsuhashi, Masato Kawakubo, Tadashi Hyodo, Yuki Murata, Masatoshi Kawahata, Kentaro Yamaguchi, Shuji Yasuike

**Affiliations:** 1 School of Pharmaceutical Sciences, Aichi Gakuin University, 1-100 Kusumoto-cho, Chikusa-ku, Nagoya 464-8650, Japan; m-matsu@dpc.agu.ac.jp (M.M.); yuki325first@yahoo.co.jp (Y.M.); masato09224@icloud.com (M.K.); y-murata@dpc.agu.ac.jp (Y.M.); 2 Pharmaceutical Sciences at Kagawa Campus, Tokushima Bunri University, 1314-1 Shido, Sanuki, Kagawa 769-2193, Japan; s170331@stu.bunri-u.ac.jp (T.H.); kawahatam@ac.shoyaku.ac.jp (M.K.); kyamaguchi@kph.bunri-u.ac.jp (K.Y.)

**Keywords:** antimony, stibole, molecular structure, low-temperature luminescence, phosphorescence

## Abstract

The expectation that antimony (Sb) compounds should display phosphorescence emissions based on the “heavy element effect” prompted our interest in the introduction of antimony to a biaryl as the bridging atom in a fused heterole system. Herein, the synthesis, molecular structures, and optical properties of novel benzene-fused heteroacenes containing antimony or arsenic atoms are described. The stiboles and arsole were prepared by the condensation of dibromo(phenyl)stibane or dichloro(phenyl)arsine with dilithium intermediates derived from the corresponding dibromo compounds. Nuclear magnetic resonance (NMR) spectroscopy and X-ray crystal analysis revealed that the linear pentacyclic stibole was highly symmetric in both the solution and crystal states. In contrast, the curved pentacyclic stibole adopted a helical structure in solution, and surprisingly, only M helical molecules were crystallized from the racemate. All synthesized compounds produced very weak or no emissions at room temperature or in the solid state. In contrast, the linear penta- and tetracyclic stiboles exhibited clear phosphorescence emissions in the CHCl_3_ frozen matrix at 77 K under aerobic conditions.

## 1. Introduction

**Figure 1:**
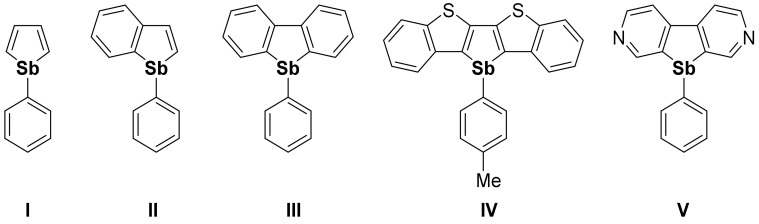
Related stiboles.

**Scheme 1:**
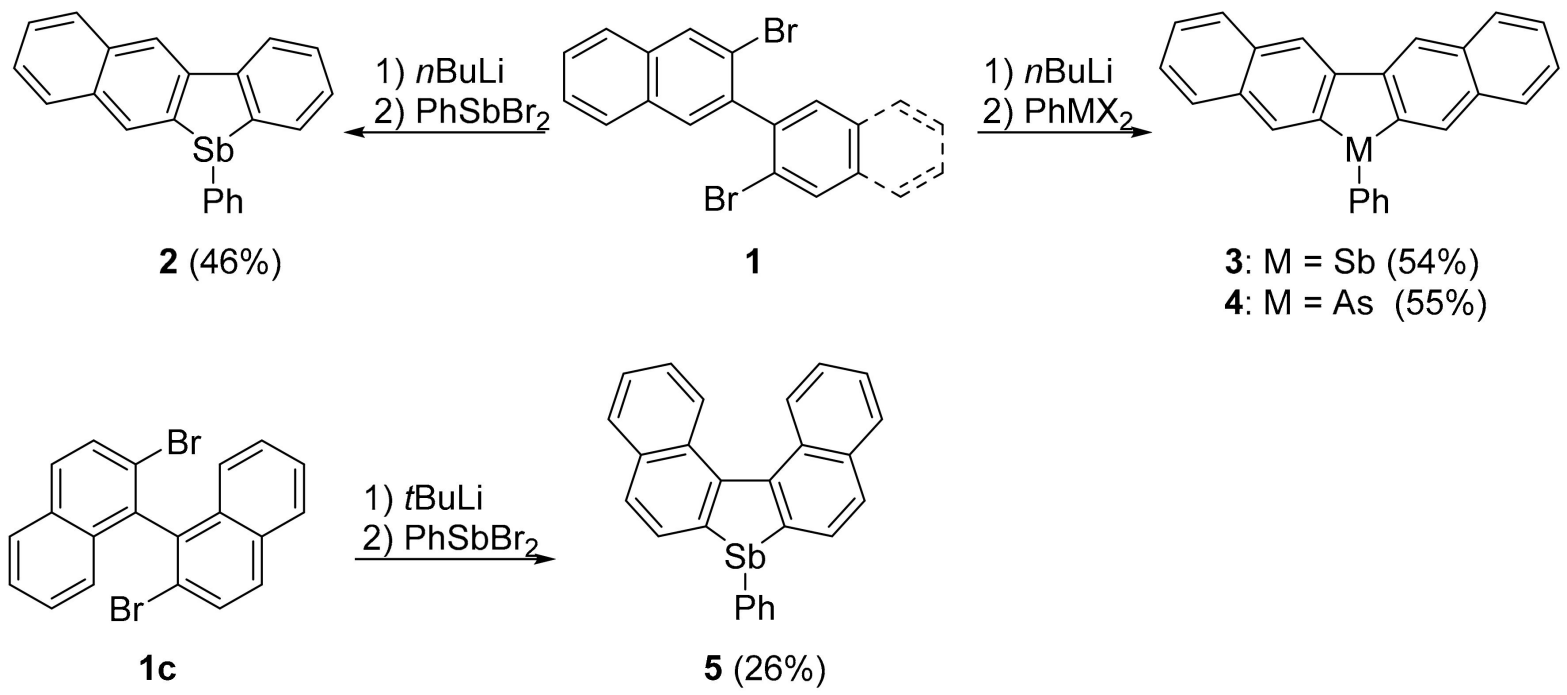
Synthetic stiboles and arsole.

The development of novel π-conjugated systems and the elucidation of their optical properties to realize desired behaviors and functionalities are of current importance in the field of organic optoelectronic materials. The introduction of a group 15 elements such as phosphorus to a biaryl, as a bridge to form a fused heterole system, is of particular interest because it can further enhance conjugation due to its electronic effects [1]. For example, five-membered phospholes and their derivatives have been extensively studied as basic units of functional materials with luminescent, semiconducting, and sensing properties [2,3,4,5,6]. Arsoles, containing the homologous element, arsenic, have been reported as luminescent substances that are less likely to be oxidized than phospholes, although toxicity is a concern [7,8,9,10,11]. Antimony, another homologue located in the fifth period of group 15 elements in the periodic table, is expected to impart phosphorescence similarly to bismuth and tellurium based on the “heavy element effect”, in contrast to lighter elements such as phosphorus [12,13,14,15,16]. Furthermore, since antimony compounds such as sodium stibogluconate (Pentostam) are used as first-line drugs for leishmaniasis [17] and are less toxic than bismuth compounds [18,19,20]; they have also garnered attention in the field of biology [21]. However, five-membered heterocyclic compounds containing antimony, known as stiboles, have received little attention with respect to their synthesis and spectrochemical behavior, including luminescence. In 1985, monocyclic stibole **I** (Figure 1) was synthesized by Ashe III et al., but it was unstable and resinified at room temperature under inert atmosphere [22]. The addition of fused aromatic rings effectively stabilizes **I**, such that the isolation of bicyclic benzo[*b*]stibole **II** and tricyclic dibenzo[*b,d*]stibole **III** has been reported [23,24,25]. The functions of benzostibole derivatives, such as the optical resolution of central asymmetry on antimony [26] and anion sensing, are being clarified [27,28]. In 2012, Ohshita et al. reported the synthesis of benzothiophene-fused pentacyclic stibole **IV** {1-(4-methylphenyl)-2,2′-di(benzo[*b*]thieno)stibolein} in low yield; however, almost no luminescence was observed at room temperature [29]. Subsequently, they reported both the synthesis of pyridine-fused tricyclic stibole **V** (9-phenyl-stibolo [2,3-*c*:5,4-*c*′]dipyridine) and its phosphorescence at low temperature. They also revealed that the copper complex derived from stibole **V** and Cu_2_I_2_(PPh_3_)_3_ displayed phosphorescence at room temperature [30]. We reported the synthesis and structural study of optically active 7-(*p*-tolyl)dinaphtho[2,1-*b*:1′,2′-*d*]stibole (7-Tol-DNSb) and revealed the dynamic behavior of the binaphthyl skeleton based on the C_2_ symmetry axis [31]. These results show that the synthesis, structural analysis, and optical properties of the parent compound, stibole, are important for the subsequent creation of functional devices. However, there are no reports of linearly extended π-conjugated tetracyclic or pentacyclic stiboles condensed with benzene rings. Here, we present the synthesis of 5-phenylbenzo[*b*]naphtho[2,3-*d*]stibole (**2**), 6-phenyldinaphtho[2,3-*b*:2′,3′-*d*]stibole (**3**), and 7-phenyldinaphtho[2,1-*b*:1′,2′-*d*]stibole (**5**) (Scheme 1) and explore the influence of the fused benzene rings on the optical properties including the phosphorescence of polycyclic benzostiboles.

## 2. Results and Discussion

### 2.1. Synthesis

The structures and synthetic routes for the novel heteroacenes prepared in this study are shown in Scheme 1. The cyclization precursors, 2-bromo-3-(2-bromophenyl)naphthalene (**1a**) and 2,2′-dibromo-3,3′-binaphthyl (**1b**), were synthesized using a modification of a previously reported procedure [32] that involved the SuzukiMiyaura cross-coupling of 3-bromo-2-naphthylboronic acid with 2-bromoiodobenzene or 3-bromo-2-iodonaphthalene, respectively. Sequential treatment of dibromo compound **1a** with *n*-butyllithium in dry tetrahydrofuran at −78 °C followed by dibromo(phenyl)stibane resulted in ring closure, giving the desired product **2** in 46% yield. Compound **3** was obtained in 54% yield from 3,3′-dibromo-2,2′-binaphthyl (**1b**) using a similar procedure. This system also afforded the analogous group 15 arsenic compound **4** in 55% yield, using dichloro(phenyl)arsine as the electrophilic reagent. We previously reported the synthesis and molecular structure of a stibohelicene, 7-Tol-DNSb, in which the positions of the fused benzene rings were different from those in **2** [31]. However, the optical properties of that compound had no mentions at all. To further probe those properties, 7-phenyldinaphtho[2,1-*b*:1′,2′-*d*]stibole (**5**) was prepared from 2,2′-dibromo-1,1′-binaphthyl (**1c**).

### 2.2. Structure Analysis

The molecular structures of compounds **2**–**5** were confirmed spectroscopically (^1^H-and ^13^C-NMR, and MS). In the ^1^H- and ^13^C-NMR spectra of linear pentacyclic stibole **3**, all the corresponding aromatic protons and carbons on the two naphthalene rings were equivalent. These results show that stibole **3** has a symmetric structure in CDCl_3_ solution. Pentacyclic arsole **4** also revealed a symmetrical structure in solution. In contrast, in the ^13^C-NMR spectrum of stibole **5**, which is an isomer of **3**, all the carbon signals in the two naphthalene rings were chemically nonequivalent. This result suggests that **5** adopts a curved helical structure similar to the known *Sb*-tolyl derivative [31].

**Figure 2:**
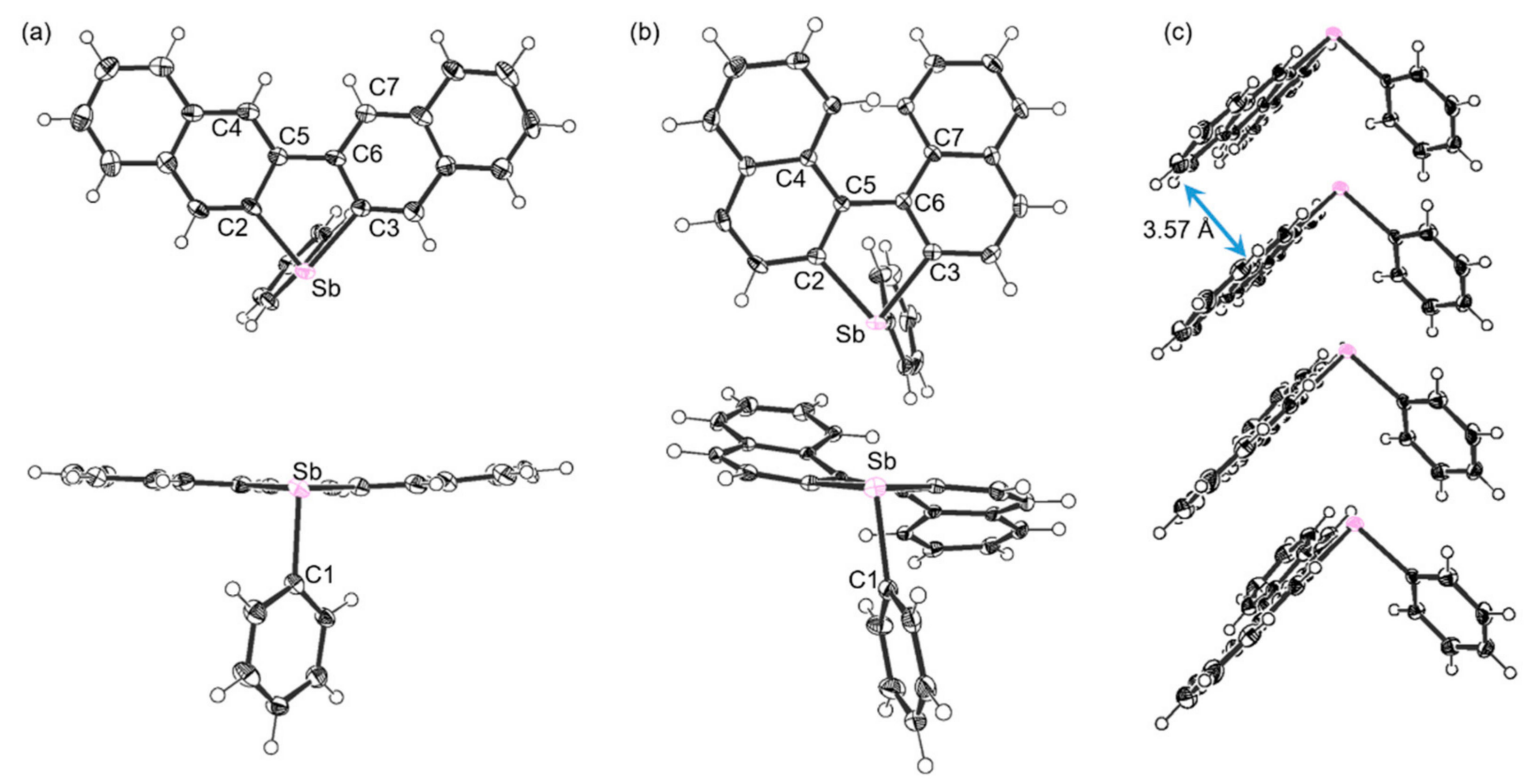
Crystal structures of (**a**) **3** and (**b**) **5**, and (**c**) stacking structure of **3**.

All the obtained heteroacenes **2**–**5** were colorless crystalline solids. Single crystals of stiboles **3** and **5** suitable for X-ray analysis were obtained by repeated recrystallization from benzene/hexane or Et_2_O/hexane as the solvent. Unfortunately, single crystals of **2** and **4** could not be obtained. The molecular structures of pentacyclic stiboles **3** and **5** obtained from single-crystal X-ray diffraction (SCXRD) analysis are illustrated in Figure 2, and selected bond lengths and angles are summarized in Table 1. The structure obtained for linear stibole **3** reveals naphthalene wings that are slightly bent. The angle between each naphthalene ring, defined by ten carbon atoms, is 8.05°, and the dihedral angle of C(4)–C(5)–C(6)–C(7) is 0.0(13)°. The C–Sb bond lengths are 2.155–2.182 Å, the C(Ph)–Sb–C bond angles are 92.7(3) and 94.6(3)°, and the interior C–Sb–C angle in the stibole ring is 81.0(3)°. The sum of the bond angles around the antimony atom is 268.3°. The antimony center is highly pyramidalized, and the Ph substituent is situated nearly perpendicular to the stibole plane. In the packing structure, π-π stacking interactions are observed along the *a* axis. The interplanar distances between adjacent dinaphthostiboles are 3.57 Å (Figure 2c). In contrast, in the case of curved stibole **5**, the C(4)–C(5)–C(6)–C(7) dihedral angle is 36.3(3)°, and a helical structure is adopted to prevent steric strain. Surprisingly, the crystallization of racemic stibole **5** produced optically active crystals consisting of only the M helical structure, that is, spontaneous resolution occurred during crystallization. Because the homochiral association event involves a large entropy loss, it has been estimated that only 5–10% of organic racemates exist as conglomerates [33,34]. To the best of our knowledge, this is the first example of the spontaneous resolution of a stibole derivative. In contrast, 7-Tol-DNSb formed a racemic crystal containing both enantiomers in equal proportions, specifically, it did not resolve spontaneously [31]. The bond lengths and angles around the antimony atom in **5** are approximately the same as in linear stibole **3**. Furthermore, similar bond lengths and angles have been observed among previously reported stiboles, including benzostiboles [27,35], dipyridostibole [30], and 7-tolyldinaphthostibole [31].

### 2.3. Optical Properties

**Figure 3:**
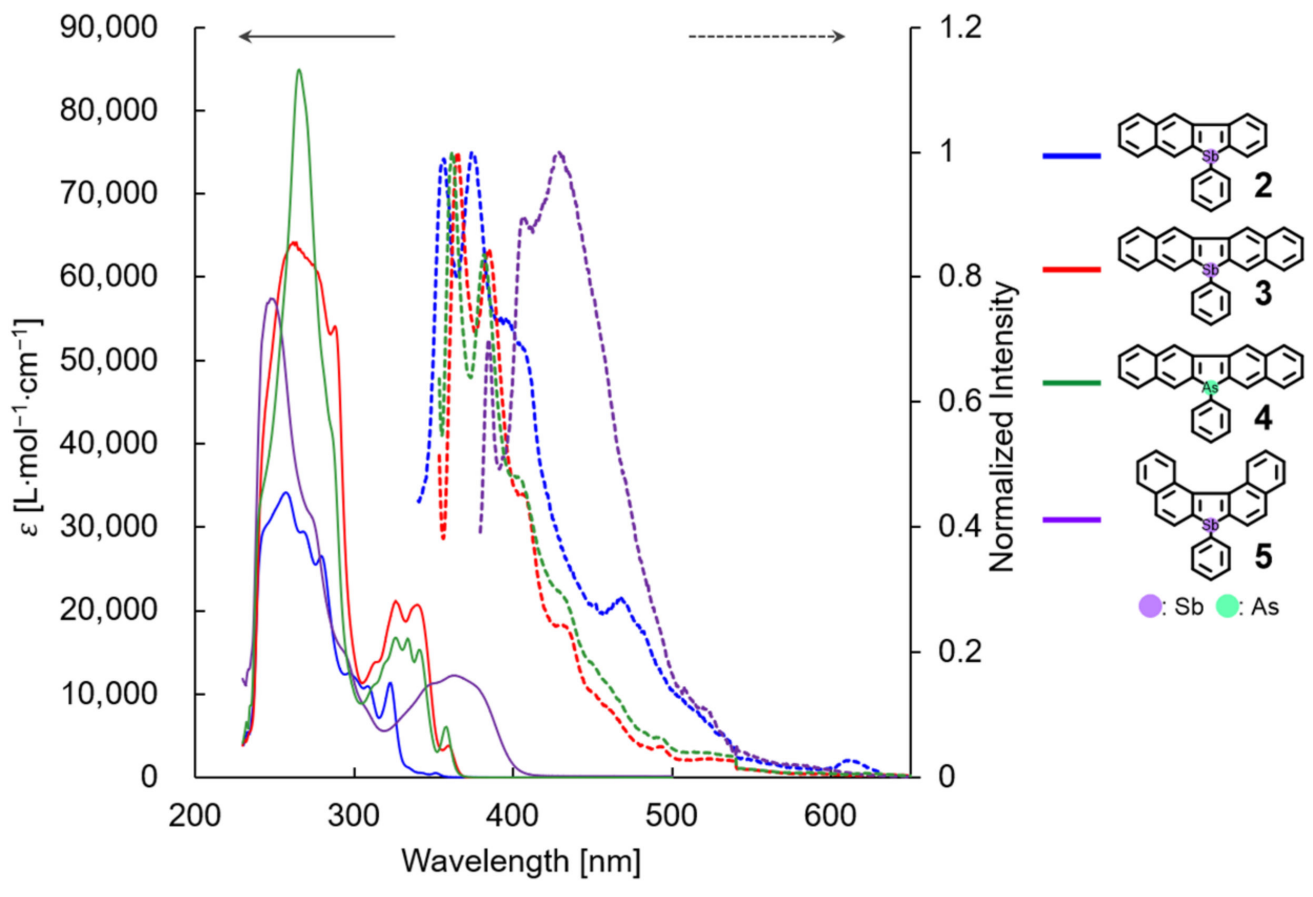
Absorption (solid lines) and normalized emission (dashed lines) spectra at room temperature of stiboles **2** (blue), **3** (red), **4** (green) and **5** (purple) in CHCl_3_. Excitation at 320 (for **2**), 340 (for **3** and **4**), and 360 nm (for **5**).

The photophysical properties of the synthesized stiboles and related compounds were evaluated, and the corresponding data are shown in Table 2 and Figure 3. The absorption maxima (*λ*_max_) of stiboles **2** and **3** are observed at 323 and 360 nm, respectively. Since the *λ*_max_ of bicyclic benzostibole **II** is 313 nm, the absorption band is red-shifted as the number of fused benzenes increases, reflecting the extended π-conjugation. Conversely, dinaphtho[2,1-*b*:1′,2′-*d*]stibole **5** has a broad absorption band at 363 nm. It is known that the *λ*_max_ of [*n*]helicenes generally occurs at lower wavelengths than the corresponding linear-type poly(acene)s [36]. In contrast, the wavelengths of peak top positions of linear stibole **3** and curved stibole **5** are the same. Surprisingly, the absorption edge of **5** reaches over 400 nm and is observed in a longer wavelength region than **3**. Arsole **4** (*λ*_max_ at 358 nm) exhibits an absorption spectrum similar to that of stibole **3**, which indicates that the influence of the bridging heteroatom composing the five-membered ring is small.

**Figure 4:**
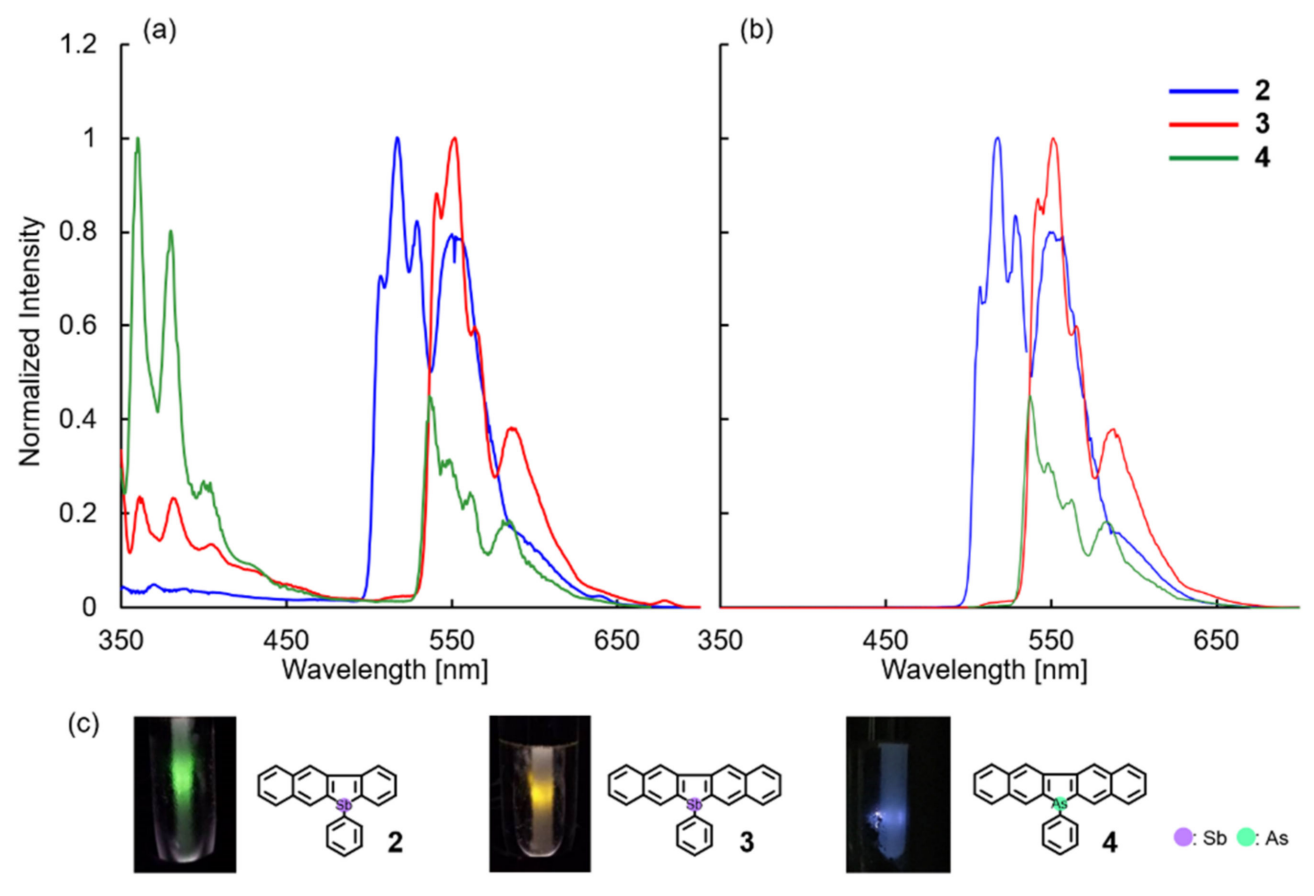
(**a**) Normalized fluorescence and (**b**) phosphorescence spectra of **2** (blue), **3** (red), and **4** (green) in CHCl_3_ at 77 K. Excitation at 320 (for **2**) and 340 nm (for **3** and **4**). Gating parameters for phosphorescence (delay, 27 ms). (**c**) Photo of the solution of **2**–**4** in CHCl_3_ at 77 K under UV irradiation (320 nm for **2** and 340 nm for **3** and **4**).

The fluorescence spectra of stiboles **2**, **3**, and **5** and arsole **4** at room temperature show weak emissions with quantum efficiencies of 3% or less. This effect can be attributed to the presence of heavy atoms that promote an effective intersystem-crossing process. In the CHCl_3_ frozen matrix at 77 K under aerobic conditions, however, stiboles **2** and **3** display apparent fluorescence and phosphorescence emissions. Figure 4 shows the fluorescence spectra (a) and phosphorescence spectra (b), isolated by means of gated detection, with a delay of 27 ms, for the compounds. These spectra clearly reveal emission bands that are composed of weaker fluorescence at 350–400 nm and stronger phosphorescence at 500–650 nm. The measured phosphorescence lifetimes are of the order of 40 ms. On the other hand, arsole **4** shows both fluorescence and phosphorescence emissions under the same conditions, but the contribution from phosphorescence (530–650 nm) is less than that from fluorescence (350–420 nm). Further, its phosphorescence lifetime is much longer than those of the stiboles (205 ms). In photographs at 77 K under UV irradiation, stiboles **2** and **3** clearly exhibit green and yellow phosphorescence emissions, respectively, whereas a blue fluorescence emission is displayed for arsole **4** (Figure 4c). However, a weak emission is observed for the helical-type stibole **5**, even at 77 K (Figure S1, Supplementary Materials). Unfortunately, none of the compounds were emissive in the crystal state.

## 3. Materials and Methods

### 3.1. General Information

Melting points were taken on a Yanagimoto micro melting point hot-stage apparatus (MP-S3) and are not corrected. ^1^H-NMR (400 MHz, TMS: δ = 0.00 ppm as an internal standard) and ^13^C-NMR (100 MHz, CDCl_3_: δ = 77.00 ppm as an internal standard) spectra were recorded on JEOL ECZ-400S spectrometer (JEOL Ltd., Tokyo, Japan) in CDCl_3_. ESI mass spectra were measured on an Agilent Technologies 6230 LC/TOF mass spectrometer (Agilent Technologies Japan, Ltd., Tokyo, Japan). IR spectra were recorded on a SHIMADZU FTIR-8400S spectrometer (SHIMADZU CORPORATION, Kyoto, Japan) and are reported in frequency of absorption (cm^−1^). Only selected IR peaks are reported. UV/Vis spectra were recorded at room temperature on a HITACHI U-2800A spectrophotometer (Hitachi High-Tech Corporation, Tokyo, Japan) (**2**: C = 5.2 × 10^−5^, **3**: C = 4.7 × 10^−5^, **4**: C = 2.7 × 10^−5^, and **5**: C = 4.5 × 10^−5^ M in CDCl_3_) and fluorescence spectra on a JASCO FP-8300 luminescence spectrometer (JASCO Corporation, Tokyo, Japan) (**2**: C = 2.1 × 10^−6^, **3**: C = 1.6 × 10^−6^, **4**: C = 1.9 × 10^−6^, and **5**: C = 2.8 × 10^−6^ M in CDCl_3_). All chromatographic separations were accomplished with Silica Gel 60N (Kanto Chemical Co., Inc., Tokyo, Japan). Thin-layer chromatography (TLC) was performed with Macherey–Nagel Pre-coated TLC plates Sil G25 UV_254_. Most of the reagents were used without further purification unless otherwise specified.

Known cyclization precursors **1b** [32] and **1c** [31] were prepared according to the reported procedures, and spectroscopic data are in accordance with the literature.

### 3.2. Preparation and Characterization of Novel Compounds

#### 3.2.1. 2-Bromo-3-(2-bromophenyl)naphthalene (**1a**)

A solution of 2-bromoiodobenzene (2.83 g, 10 mmol) and tetrakis(triphenylphosphine)- palladium(0) (1.04 g, 0.90 mmol, 9 mol%) in dry toluene (80 mL) were stirred for 1 h under Ar atmosphere. Then a solution of 3-bromo-2-naphthylboronic acid (3.76 g, 15 mmol, 1.5 eq.) in dry ethanol and sodium thiosulfate aqueous (2.45 M, 25 mL, 60 mmol, 6 eq.) were added to the reaction mixture, and reflux at 85 °C. After 2.5 h, the reaction mixture was diluted with CH_2_Cl_2_ (150 mL) and water (100 mL) at 0 °C. The phases were separated and the aqueous layer was extracted with CH_2_Cl_2_ (50 mL × 3). The combined organic layer was washed with sat. sodium thiosulfate aqueous (30 mL × 3), dried over anhydrous magnesium sulfate, filtered, and concentrated under reduced pressure. The residue was purified by column chromatography using *n*-hexane as eluent to give **1a** as a colorless plate (3.36 g, 93%),m.p.109–112 °C (benzene/ethanol). ^1^H-NMR (400 MHz, CDCl_3_) δ: 8.18 (s, 1H), 7.83–7.79 (m, 2H), 7.72 (s, 1H), 7.69 (d, *J* = 8.3 Hz, 1H), 7.56–7.49 (m, 2H), 7.40 (t, *J* = 7.3 Hz, 1H), 7.33–7.24 (m, 2H). ^13^C-NMR (100 MHz, CDCl_3_) δ: 142.0 (C), 139.5 (C), 133.8 (C), 132.5 (CH), 132.0 (C), 131.2 (CH), 131.0 (CH), 129.8 (CH), 129.4 (CH), 128.0 (CH), 127.10 (CH), 127.05 (CH), 126.8 (CH), 126.6 (CH), 124.0 (C), 121.3 (C). FTIR (KBr): 3051, 1423, 1424, 876, 746 cm^−1^. LRMS (EI) *m*/*z*: 362 ([M]^+^, 99%), 283 (30%), 202 (100%), 83 (73%). HRMS: *m*/*z* [M]^+^ calculated for C_16_H_10_Br_2_: 361.9129. found: 361.9131.

#### 3.2.2. 5-Phenylbenzo[*b*]naphtho[2,3-*d*]stibole (**2**)

A solution of *n*-BuLi (1.61 M solution in hexane, 4.5 mL, 7.2 mmol, 2.1 eq.) was added dropwise to a solution of 2-bromo-3-(2-bromophenyl)naphthalene (**1a**) (1.24 g, 3.4 mmol) in dry THF (40 mL) at −78 °C under Ar atmosphere. After 1.5 h, dibromo(phenyl)stibane (1.60 g, 4.5 mmol, 1.3 eq.) was added to the reaction mixture and the resulting mixture was stirred for 1 h. The reaction mixture was diluted with CH_2_Cl_2_ (40 mL) and water (40 mL) at 0 °C. The phases were separated, and the aqueous layer was extracted with CH_2_Cl_2_ (50 mL × 3). The combined organic layer was washed with water (30 mL × 3), dried over anhydrous magnesium sulfate, filtered, and concentrated under reduced pressure. The residue was purified by column chromatography using *n*-hexane/CH_2_Cl_2_ (10:1) as eluent to give **2** as a colorless needle (621 mg, 46%). m.p. 118–120 °C (CH_2_Cl_2_/*n*-hexane). ^1^H-NMR (400 MHz, CDCl_3_) δ: 8.33 (s, 1H), 8.18 (s, 1H), 8.10 (d, *J* = 7.9 Hz, 1H), 7.86 (d, *J* = 7.6 Hz, 1H), 7.75 (t, *J* = 8.6 Hz, 2H), 7.49–7.42 (m, 3H), 7.34–7.29 (m, 3H), 7.17–7.10 (m, 3H). ^13^C-NMR (100 MHz, CDCl_3_) δ: 149.7 (C), 147.4 (C), 145.1 (C), 142.7 (C), 138.7 (C), 135.6 (CH), 135.4 (CH), 135.2 (CH), 133.6 (C), 133.3 (C), 129.0 (CH), 128.7 (CH), 128.49 (CH), 128.45 (CH), 128.1 (CH), 127.5 (CH), 126.5 (CH), 126.0 (CH), 123.8 (CH), 121.7 (CH). FTIR (KBr): 3048, 1429, 880, 772, 733, 692 cm^−1^. LRMS (EI) *m*/*z*: 400 ([M]^+^, 100%), 323 (50%), 279 (73%), 202 (55%). HRMS: *m*/*z* [M]^+^ calculated for C_22_H_13_Sb: 400.0212. Found: 400.0209.

#### 3.2.3. 6-Phenyldinaphtho[2,3-*b*,2′,3′-*d*]stibole (**3**)

A solution of *n*-BuLi (1.54 M solution in hexane, 3 mL, 4.8 mmol, 2.4 eq.) was added dropwise to a solution of 3,3′-dibromo-2,2′-binaphthyl (**1b**) (823 mg, 2.0 mmol) in dry THF (30 mL) at −78 °C under Ar atmosphere. After 1 h, dibromo(phenyl)stibane (1.07 g, 3.0 mmol, 1.5 eq.) was added to the reaction mixture and the resulting mixture was stirred for 1 h. The reaction mixture was diluted with CH_2_Cl_2_ (30 mL) and water (30 mL) at 0 °C. The phases were separated, and the aqueous layer was extracted with CH_2_Cl_2_ (30 mL × 3). The combined organic layer was washed with water (30 mL × 3), dried over anhydrous magnesium sulfate, filtered, and concentrated under reduced pressure. The residue was purified by column chromatography using *n*-hexane/CH_2_Cl_2_ (10:1) as eluent to give **3** as a colorless needle (483 mg, 54%). m.p. 227–229.5 °C (benzene/*n*-hexane). ^1^H-NMR (400 MHz, CDCl_3_) δ: 8.56 (s, 2H), 8.24 (s, 2H), 7.96 (d, *J* = 7.9 Hz, 2H), 7.80 (d, *J* = 7.6 Hz, 2H), 7.53–7.43 (m, 4H) 7.35 (d, *J* = 7.9 Hz, 2H), 7.17–7.09 (m, 3H). ^13^C-NMR (100 MHz, CDCl_3_) δ: 146.9 (C), 142.8 (C), 139.3 (C), 135.7 (CH), 135.1 (CH), 133.8 (C), 133.6 (C), 128.7 (CH), 128.5 (CH), 128.3 (CH), 127.6 (CH), 126.6 (CH), 126.1 (CH), 122.1 (CH). FTIR (KBr): 3046, 876, 745, 731, 694 cm^−1^. LRMS (EI) *m*/*z*: 450 ([M]^+^, 100%), 373 (45%), 252 (68%), 149 (28%). HRMS: *m*/*z* [M]^+^ calculated for C_26_H_17_Sb: 450.0368. Found: 450.0364.

#### 3.2.4. 6-Phenyldinaphtho[2,3-*b*,2′,3′-*d*]arsole (**4**)

A solution of *n*-BuLi (1.60 M solution in hexane, 2.3 mL, 3.6 mmol, 2.4 eq.) was added dropwise to a solution of 3,3′-dibromo-2,2′-binaphthyl (**1b**) (618 mg, 1.5 mmol) in dry THF (24 mL) at −78 °C under Ar atmosphere. After 20 min, dichloro(phenyl)arisine (0.5 mL, 3.6 mmol, 2.4 eq.) was added to the reaction mixture and the resulting mixture was stirred for 30 min. The reaction mixture was diluted with CH_2_Cl_2_ (45 mL) and water (45 mL) at 0 °C. The phases were separated, and the aqueous layer was extracted with CH_2_Cl_2_ (30 mL × 3). The combined organic layer was washed with water (30 mL × 3), dried over anhydrous magnesium sulfate, filtered, and concentrated under reduced pressure. The residue was purified by column chromatography using *n*-hexane/CH_2_Cl_2_ (4:1) as eluent to give **4** as a colorless prism (333 mg, 55%). m.p. 209–211 °C (CH_2_Cl_2_/*n*-hexane). ^1^H-NMR (400 MHz, CDCl_3_) δ: 8.48 (s, 2H), 8.19 (s, 2H), 7.93 (d, *J* = 7.8 Hz, 2H), 7.79 (d, *J* = 8.3 Hz, 2H), 7.51–7.43 (m, 4H) 7.30 (dd, *J* = 7.3, 2.0 Hz, 2H), 7.17-7.10 (m, 3H). ^13^C-NMR (100 MHz, CDCl_3_) δ: 143.5 (C), 142.8 (C), 141.5 (C), 133.8 (C), 133.6 (C), 132.0 (CH), 131.6 (CH), 128.7 (CH), 128.5 (CH), 128.4 (CH), 127.8 (CH), 126.5 (CH), 126.1 (CH), 120.9 (CH). FTIR (KBr): 3046, 874, 745, 737 cm^−1^. LRMS (EI) *m*/*z*: 404 ([M]^+^, 43%), 327 (40%), 254 (63%), 83 (100%). HRMS: *m*/*z* [M]^+^ calculated for C_26_H_17_Sb: 404.0546. Found: 404.0544.

#### 3.2.5. 7-Phenyldinaphtho[2,1-*b*,1′,2′-*d*]stibole (**5**)

A solution of 2,2′-dibromo-1,1′-binaphthyl (**1c**) (827 mg, 2.0 mmol) in dry THF (45 mL) was added dropwise to a solution of *t*-BuLi (1.60 M solution in hexane, 6.2 mL, 10 mmol, 5 eq.) in dry THF (20 mL) at −78 °C under Ar atmosphere. After 10 min, dibromo(phenyl)stibane (1.07 g, 3.0 mmol, 1.5 eq.) was added to the reaction mixture and the resulting mixture was stirred for 1 h. The reaction mixture was diluted with CH_2_Cl_2_ (30 mL) and water (30 mL) at 0 °C. The phases were separated and the aqueous layer was extracted with CH_2_Cl_2_ (30 mL × 3). The combined organic layer was washed with water (30 mL × 3), dried over anhydrous magnesium sulfate, filtered, and concentrated under reduced pressure. The residue was purified by column chromatography using *n*-hexane/CH_2_Cl_2_ (10:1) as eluent to give **5** as a colorless needle (220 mg, 24%). m.p. 142–143.5 °C (Et_2_O/*n*-hexane). ^1^H-NMR (400 MHz, CDCl_3_) δ: 7.96–7.90 (m, 5H), 7.86 (dd, *J* = 7.8, 2.4 Hz, 2H), 7.85 (d, *J* = 7.8 Hz, 1H), 7.51–7.47 (m, 2H), 7.34 (tt, *J* = 9.5, 2.0 Hz, 2H) 7.25 (dd, *J* = 7.7, 1.5 Hz, 2H), 7.15 (tt, *J* = 7.8, 1.5 Hz, 1H), 7.09 (t, *J* = 7.8 Hz, 2H). ^13^C-NMR (100 MHz, CDCl_3_) δ: 149.0 (C), 148.1 (C), 145.0 (C), 144.0 (C), 138.3 (C), 135.5 (CH), 134.7 (C), 134.3 (C), 131.9 (C), 131.7 (C), 131.6 (CH), 130.6 (CH), 128.8 (CH), 128.7 (CH), 128.5 (CH), 128.3 (CH), 128.2 (CH), 128.1 (CH), 128.0 (CH), 127.8 (CH), 125.7 (CH), 125.6 (CH), 124.53 (CH), 124.52 (CH). FTIR (KBr): 3046, 1431, 812, 750, 729 cm^−1^. LRMS (EI) *m*/*z*: 450 ([M]^+^, 99%), 371 (30%), 329 (100%), 252 (60%). HRMS: *m*/*z* [M]^+^ calculated for C_26_H_17_Sb: 450.0368. Found: 450.0371.

### 3.3. Single Crystal X-ray Diffraction Experiment

#### 3.3.1. Compound **3**

The colorless plate crystal (0.100 × 0.070 × 0.020 mm^3^), obtained from methanol/dichloromethane, was immersed in Paraton-N oil and placed in the N_2_ cold stream at 100 K. The diffraction experiment was performed in a Bruker APEX II system (Bruker, Kanagawa, Japan) (APEX II CCD detector, MoKα: λ = 0.71073 Å). Absorption correction was performed by an empirical method implemented in SADABS [37]. Structure solution and refinement were performed by using SHELXS-2014/7 and SHELXL-2014/7 [38]. 

C_26_H_17_Sb, *M*r = 451.14; orthorhombic, space group *P*2_1_2_1_2_1_, *Z* = 4, *D*_calc_ = 1.617 g·cm^−3^, *a* = 5.1377(17), *b* = 12.092(4), *c* = 29.834(10) Å, *V* = 1853.5(11) Å^3^, 16,845 measured and 3415 independent [*I* > 2σ(*I*)] reflections, 144 parameters, final *R*_1_ = 0.0383, *wR*_2_ = 0.0744, *S* = 1.081 [*I* > 2σ(*I*)]. CCDC 2041167.

All non-hydrogen atoms were refined anisotropically. The hydrogen atoms were refined isotropically on the calculated positions using a riding model (AFIX 43) with Uiso values constrained to 1.2/1.5 Ueq of their parent atoms.

#### 3.3.2. Compound **5**

The colorless prismatic crystal (0.300 × 0.300 × 0.150 mm^3^), obtained from ether/hexane, was immersed in Paraton-N oil and placed in the N_2_ cold stream at 100 K. The diffraction experiment was performed in a Bruker APEX II system (Bruker, Kanagawa, Japan) (APEX II CCD detector, MoKα: λ = 0.71073 Å). Absorption correction was performed by an empirical method implemented in SADABS [37]. Structure solution and refinement were performed by using SHELXS-2014/7 and SHELXL-2014/7 [38].

C_26_H_17_Sb, *M*r = 451.14; orthorhombic, space group *P*2_1_2_1_2_1_, *Z* = 4, *D*_calc_ = 1.563 g·cm^−3^, *a* = 8.5914(15), *b* = 9.9720(17), *c* = 22.374(4) Å, *V* = 1916.9(6) Å^3^, 17,554 measured and 3498 independent [*I* > 2σ(*I*)] reflections, 244 parameters, final *R*_1_ = 0.0114, *wR*_2_ = 0.0311, *S* = 0.985 [*I* > 2σ(*I*)]. CCDC 2041187.

All non-hydrogen atoms were refined anisotropically. The hydrogen atoms were refined isotropically on the calculated positions using a riding model (AFIX 43) with Uiso values onstrained to 1.2/1.5 Ueq of their parent atoms.

## 4. Conclusions

In this study, penta- and tetracyclic stiboles and a pentacyclic arsole were synthesized using a double lithiation method. The NMR spectra revealed that linear pentacyclic compounds **3** and **4** had highly symmetric structures in solution. In contrast, the two naphthalene rings in curved pentacyclic stibole **5** were chemically nonequivalent. X-ray analysis revealed that the naphthalene wings of linear stibole **3** were slightly bent and packing structure of **3** formed π-π stacking. In the case of curved stibole **5**, prepared as a racemate, spontaneous resolution occurred during crystallization, and the single crystal of compound **5** contained only M helical molecules. This is the first example of spontaneous resolution for a stibole derivative. All compounds displayed very weak or no emissions at room temperature or in the solid state. In contrast, linear compounds **2**–**4** showed clear phosphorescence emissions in the CHCl_3_ frozen matrix at 77 K under aerobic conditions. Further investigations are underway, including synthesize of other isomers by the different in a position of fused benzene ring and the evaluation of the physicochemical properties, such as fluorescence, phosphorescence and redox potential, by theoretical and electrochemical studies.

## Figures and Tables

**Table 1:** Selected bond lengths and angles.

		Compound 3	Compound 5
Bond length (Å)	C(1)-Sb(1)	2.182(9)	2.158(2)
	C(2)-Sb(1)	2.155(10)	2.138(2)
	C(3)-Sb(1)	2.171(8)	2.146(2)
Bond angle (°)	C(2)-Sb(1)-C(3)	81.0(3)	80.03(8)
	C(1)-Sb(1)-C(2)	92.7(3)	100.33(8)
	C(1)-Sb(1)-C(3)	94.6(3)	89.47(8)
Torsion angle (°)	C(4)-C(5)-C(6)-C(7)	0.0(13)	36.3(3)

**Table 2:** Absorption and emissions spectroscopy data ^a^.

Compd.	UV *λ*_abs_ (*ε*) (nm)	*λ*_fl_^b^	*Φ*_fl_^b,c^	*λ*_phos_^d^	*τ*_phos_^d^
		(nm)	(%)	(nm)	(ms)
**2**	268 (29500), 323 (11300)	356, 373	1.0	517, 529	40
**3**	288 (54100), 340 (20700), 360 (3700)	365, 385	0.5	551, 588	45
**4**	266 (84800), 341 (15300), 358 (6100)	362	3.0	537, 583	205
**5**	363 (12200)	406.5	0.03	n.d. ^e^	n.d. ^e^

^a^ Measured in CHCl_3_. ^b^ Excited at 320 for **2**, 340 for **3** and **4**, and 360 nm for **5**. ^c^ Measured using anthracene as a standard. ^d^ Measured at 77 K in gated detection (delay 27 ms). ^e^ Not detected.

## Data Availability

The data presented in this study are available in this article.

## Supplementary Materials

The following are available online, Figure S1: Fluorescence and phosphorescence spectra of compound **5**. ^1^H- and ^13^C-NMR spectra are available online. The crystal structures have been deposited to the CCDC with the numbers 2041167 and 2041187, and CIF files are also provided as Supplementary Materials.

## Sample Availability

Not available.
