# Supplementary material for: Synthesis, Structural Characterization, and Optical Properties of Benzene-Fused Tetracyclic and Pentacyclic Stiboles
Source: Molecules. 2021 Jan 4;26(1):222. doi: 10.3390/molecules26010222 (PMC7795936; doi:10.3390/molecules26010222)

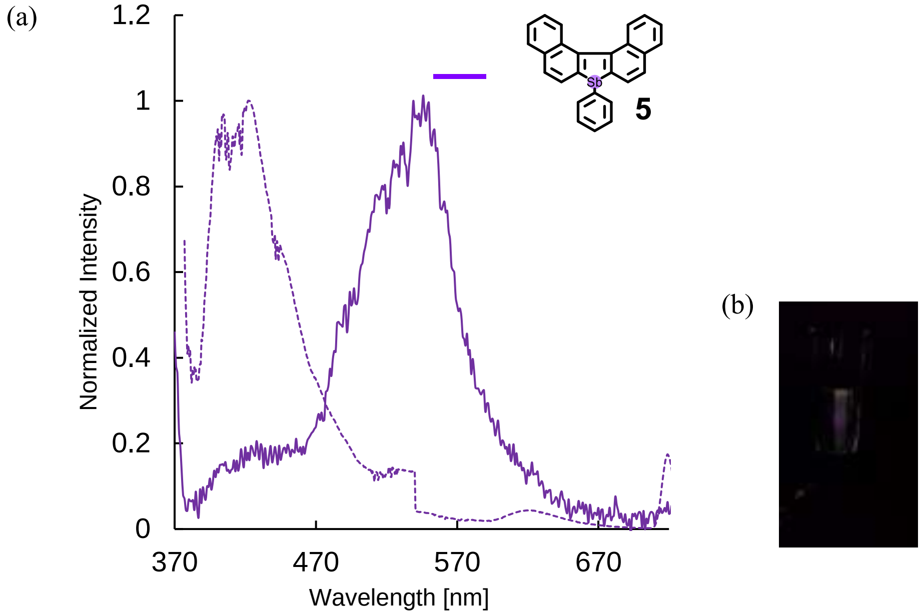

Figure S1. a) Normalized fluorescence (dashed lines) and phosphorescence (solid lines) spectra of **5** in $CHCl_3$ at 77 K. Excitation at 360 nm. Gating parameters for phosphorescence (delay, 27 ms). b) Photo of the solution in $CHCl_3$ at 77 K under UV irradiation (360 nm).

NMR spectra of novel compounds

^{1}H NMR of **1a**

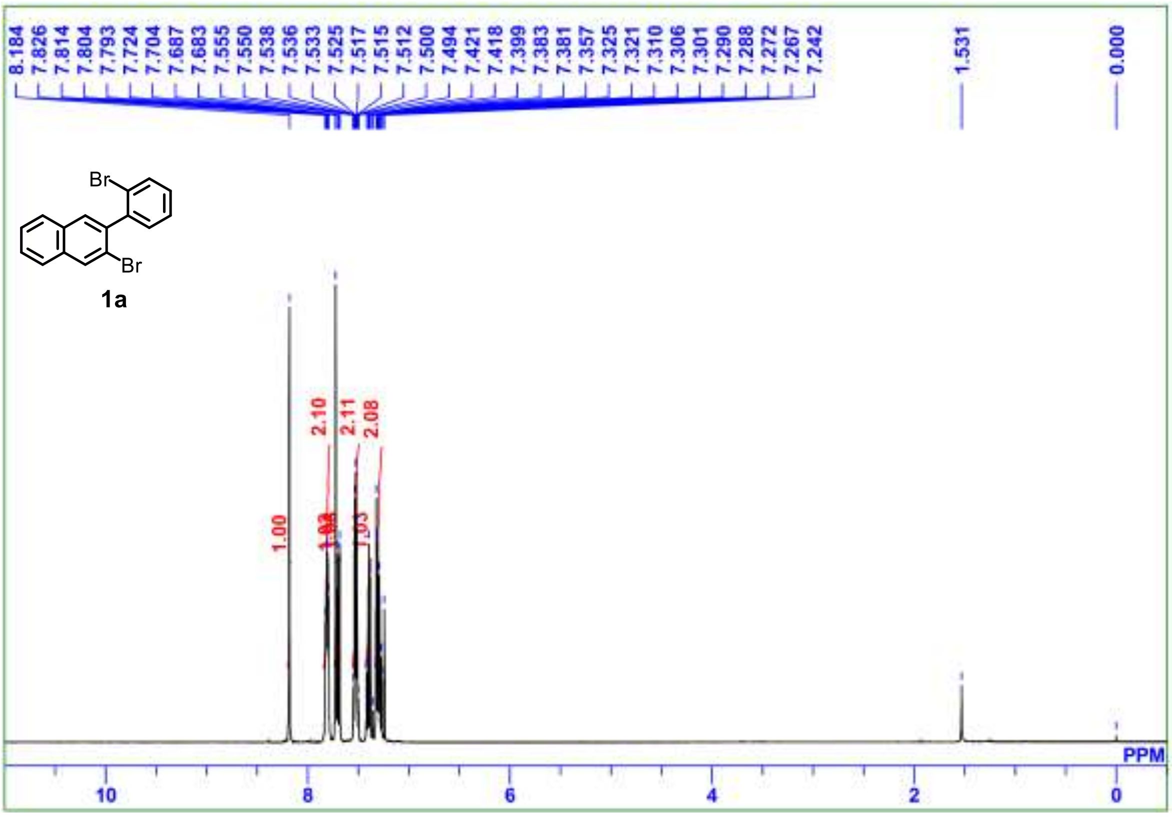

^{13}C NMR of **1a**

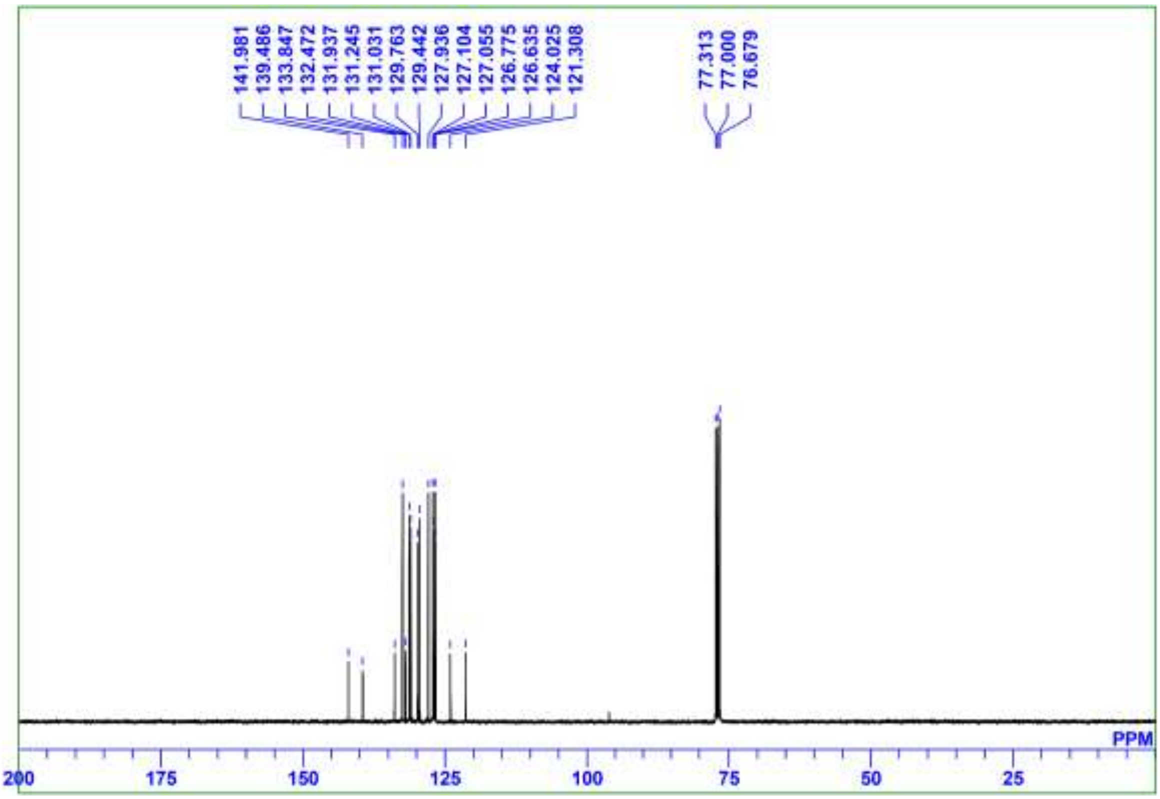

^{1}H NMR of **2**

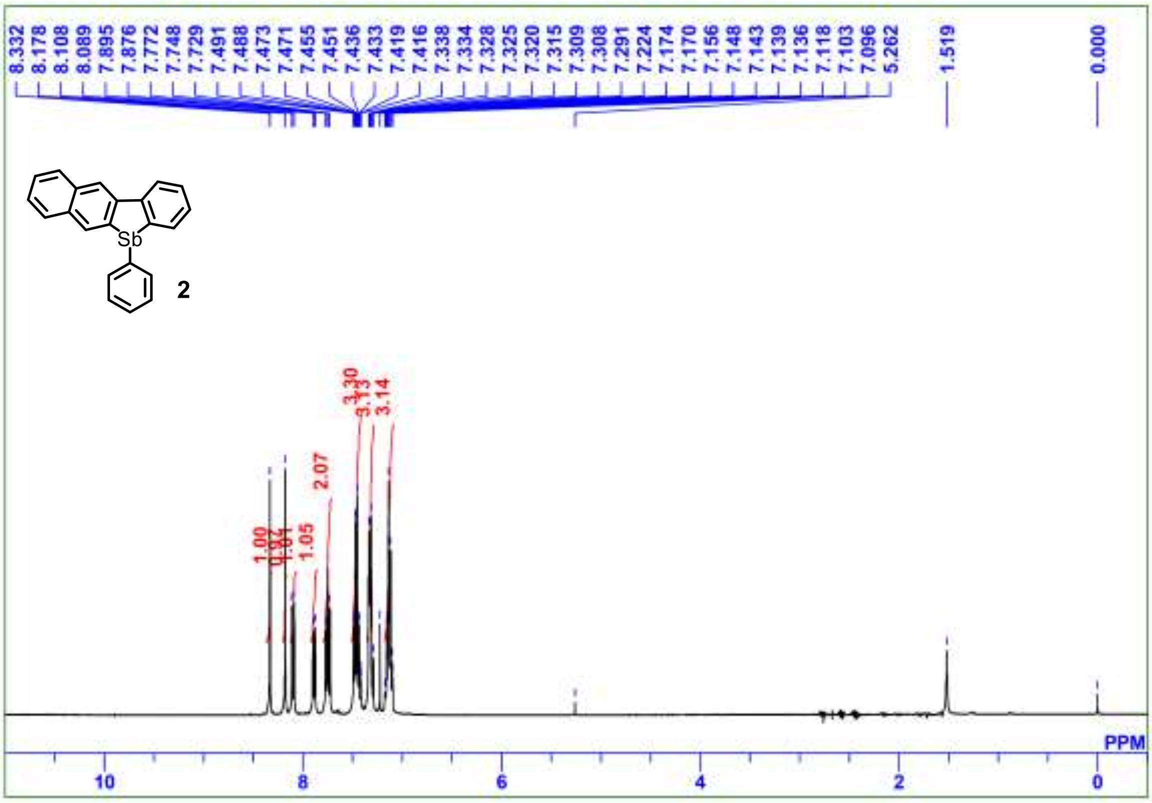

^{13}C NMR of **2**

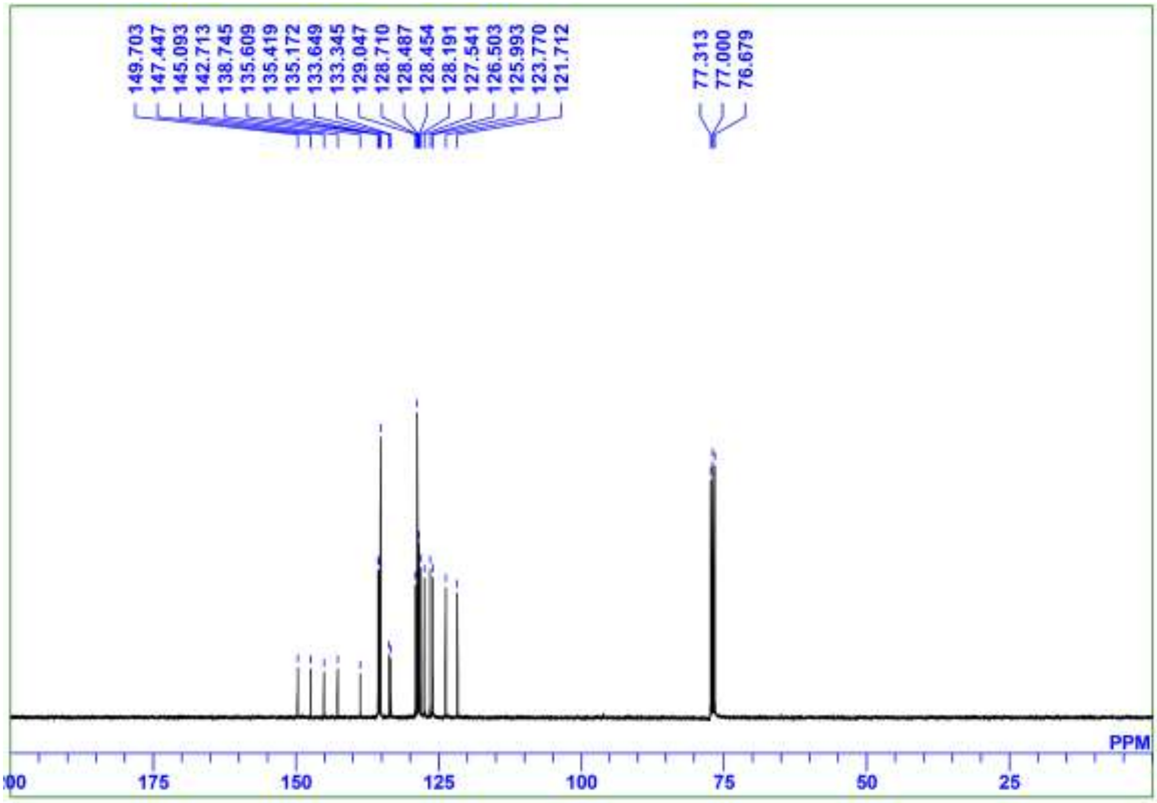

^{1}H NMR of **3**

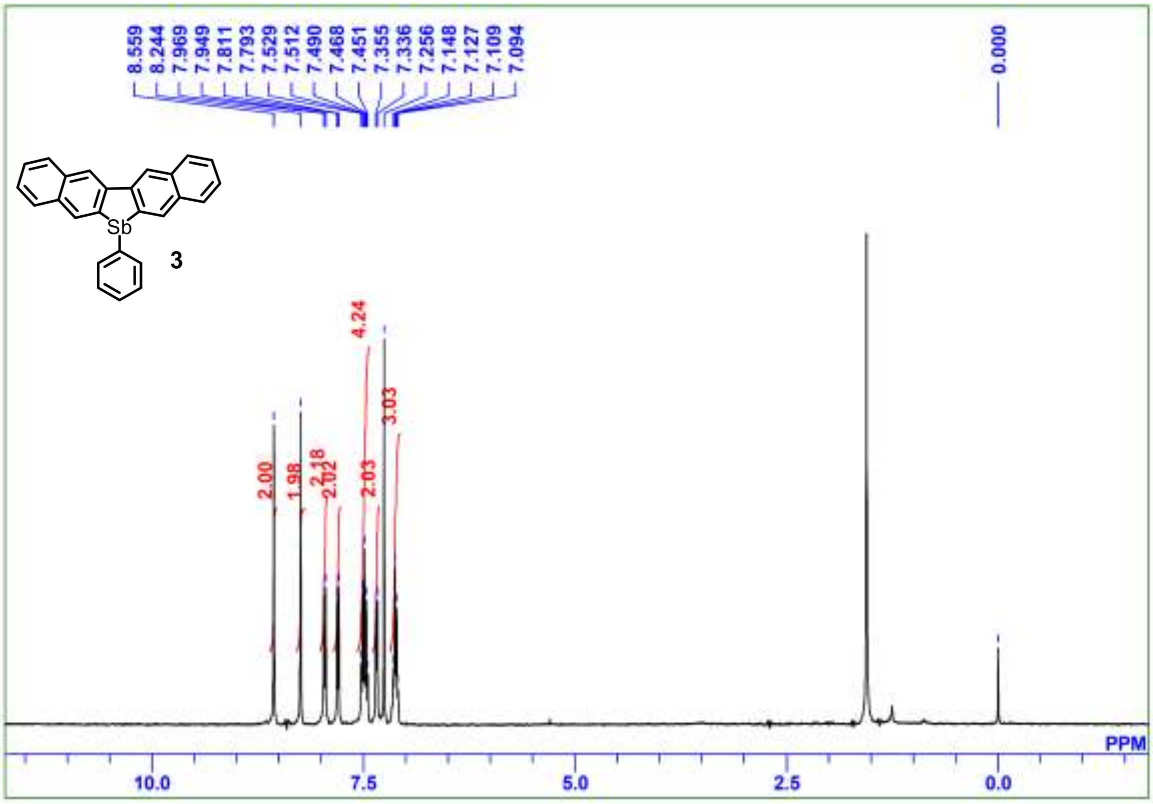

^{13}C NMR of **3**

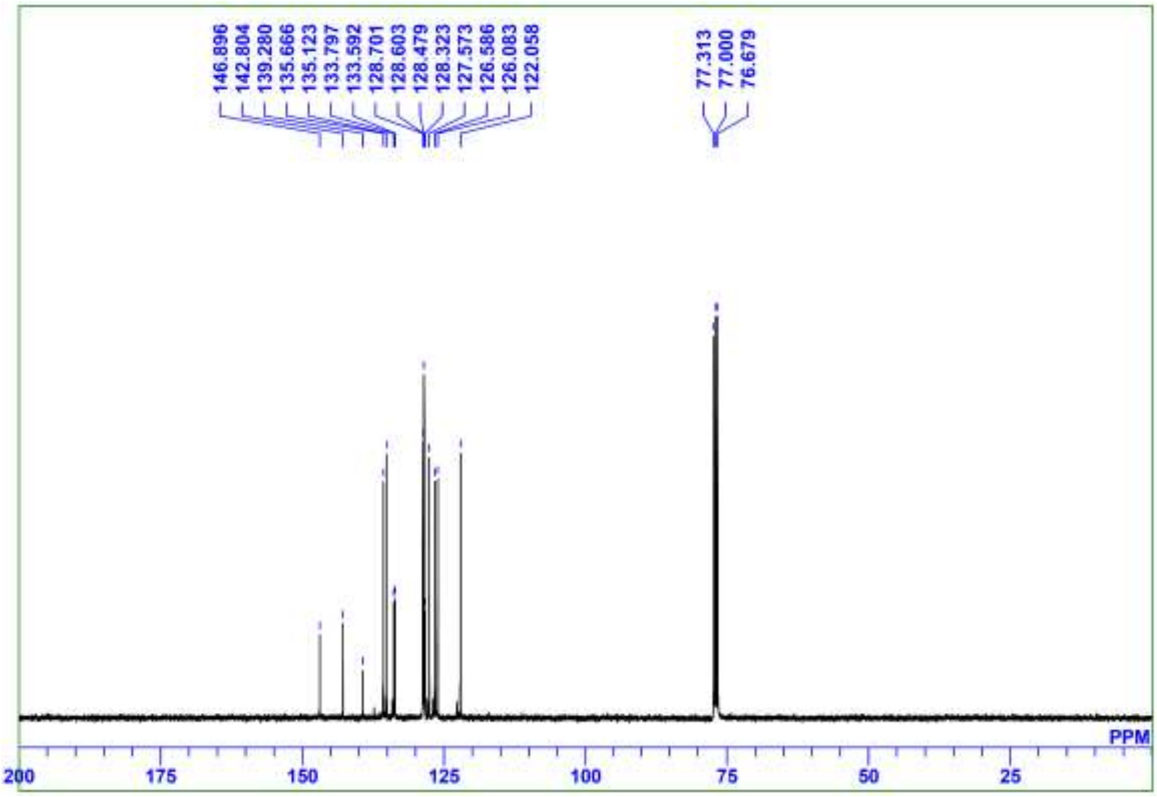

^{1}H NMR of **4**

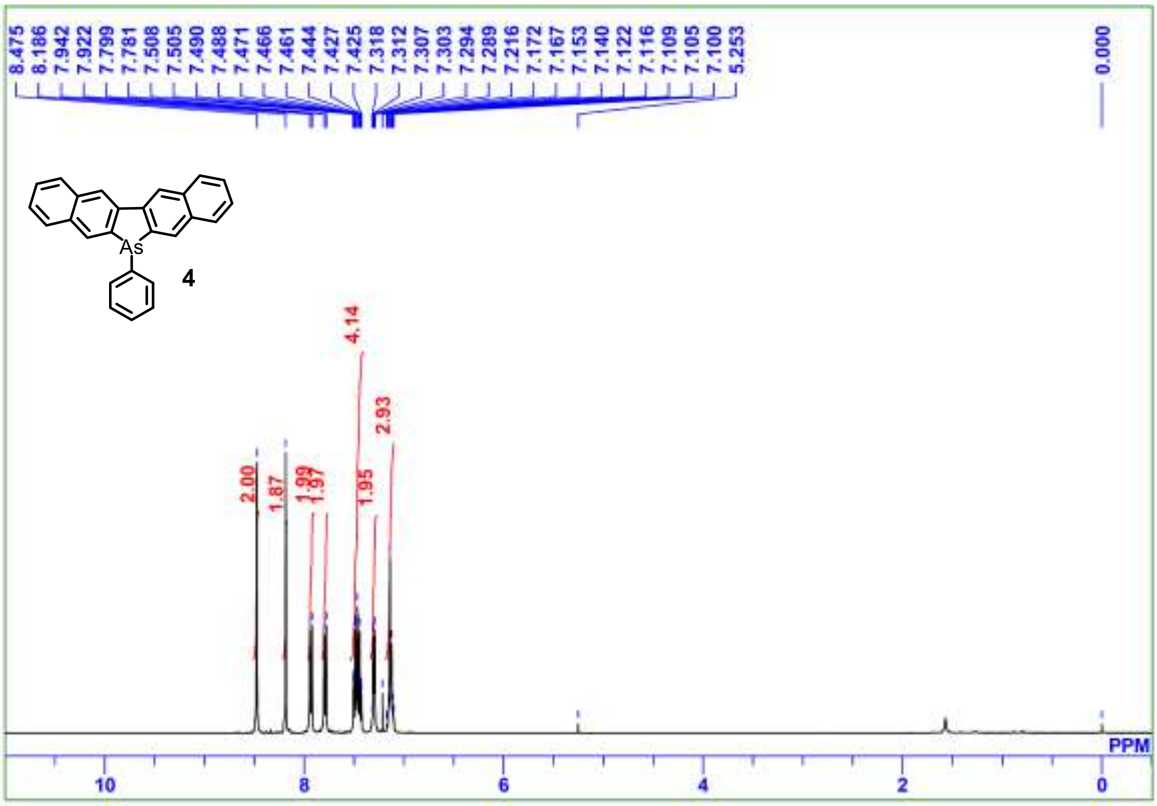

^{13}C NMR of **4**

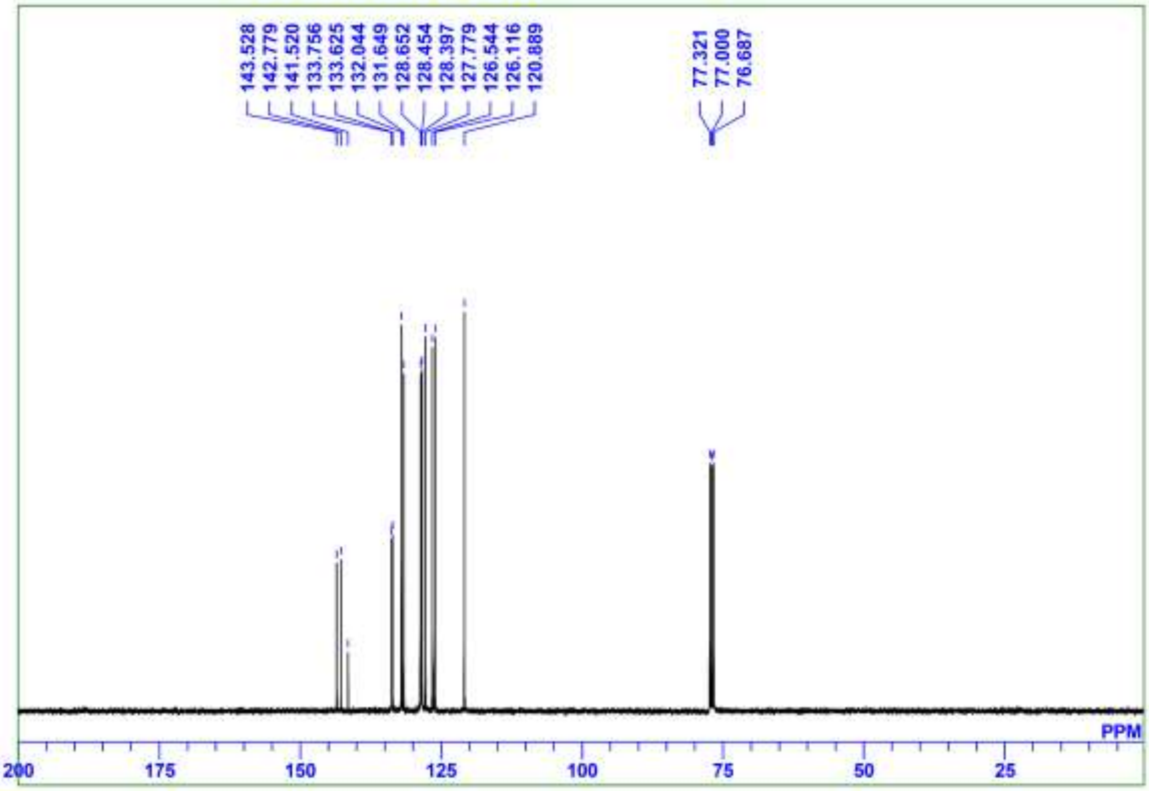

^{1}H NMR of **5**

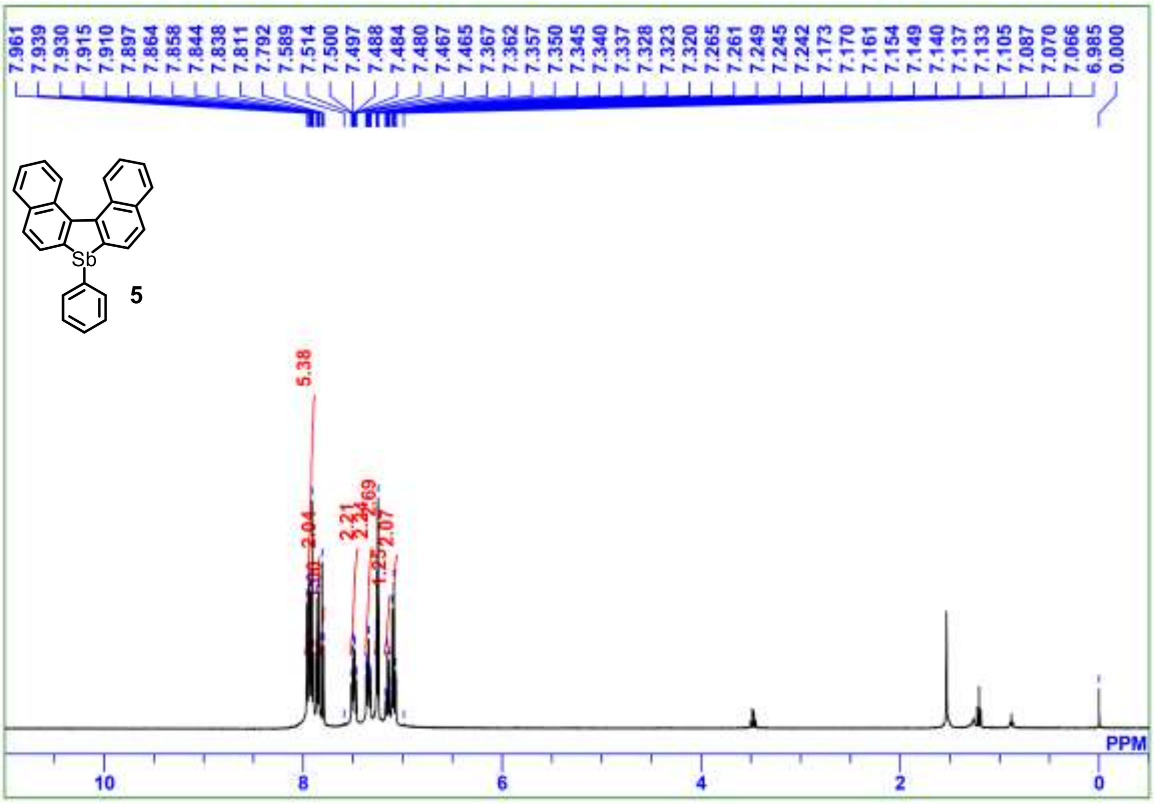

^{13}C NMR of **5**

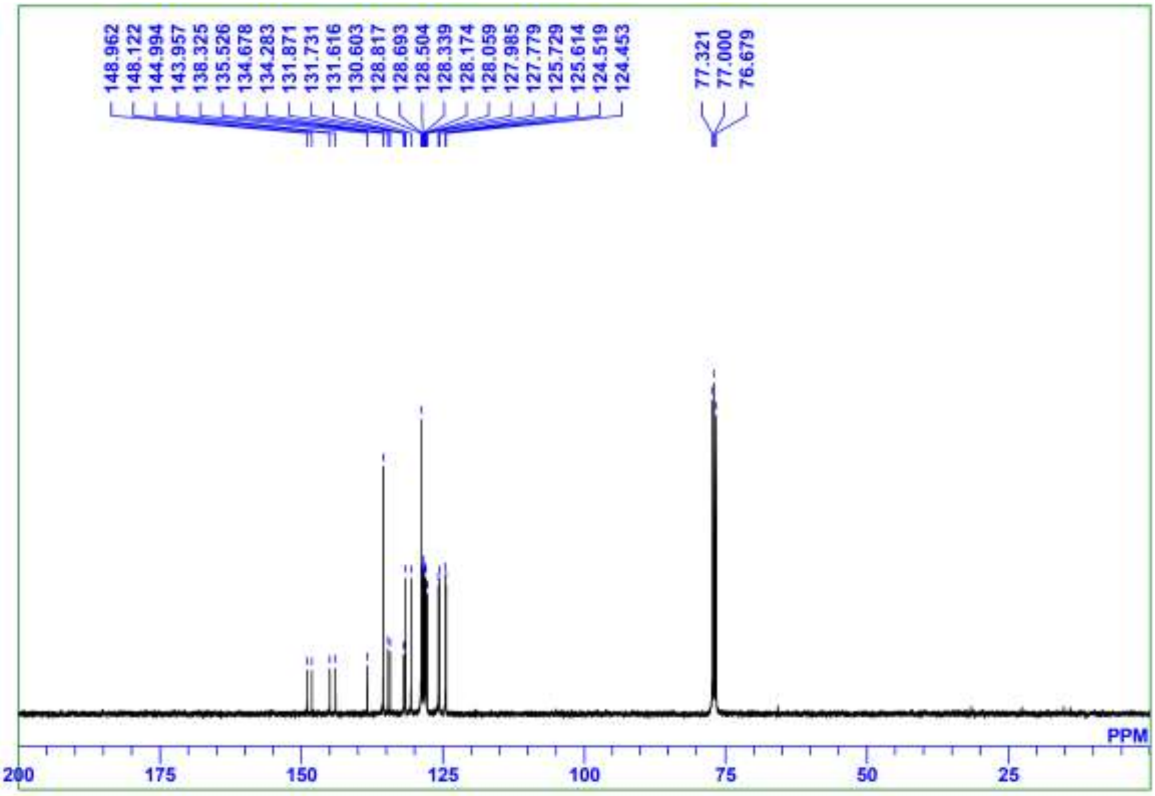

Supplement: Supplementary file 1 [file molecules-26-00222-s001.pdf]
